# Preperitoneal Fat Grafting Inhibits the Formation of Intra-abdominal Adhesions in Mice

**DOI:** 10.1007/s11605-019-04425-4

**Published:** 2019-12-10

**Authors:** Mervi Laukka, Erika Hoppela, Jemiina Salo, Pia Rantakari, Tove J. Gronroos, Katri Orte, Kaisa Auvinen, Marko Salmi, Heidi Gerke, Kerstin Thol, Emilia Peuhu, Saila Kauhanen, Pirjo Merilahti, Pauliina Hartiala

**Affiliations:** 1 Department of Plastic and General Surgery, Turku University Hospital, Turku, Finland; 2 Institute of Biomedicine, University of Turku, Turku, Finland; 3 Turku PET Centre, University of Turku, Turku, Finland; 4 Medicity Research Laboratories, University of Turku, Turku, Finland; 5 Genetics and Saske Tyks Laboratory Division, Turku University Hospital, Turku, Finland; 6 Centre for Biotechnology, University of Turku, Turku, Finland; 7 FICAN West Cancer Laboratory, Turku University Hospital and University of Turku, Turku, Finland; 8 Department of Gastrointestinal Surgery, Turku University Hospital, Turku, Finland

**Keywords:** Laparotomy, Peritoneum, Postoperative adhesions, Fat grafting, Wound healing

## Abstract

**Background:**

Adhesion formation contributes to postoperative complications in abdominal and gynaecological surgery. Thus far, the prevention and treatment strategies have focused on mechanical barriers in solid and liquid form, but these methods are not in routine use. As autologous fat grafting has become popular in treatment of hypertrophic scars because of its immunomodulatory effects, we postulated that fat grafting could also prevent peritoneal adhesion through similar mechanisms.

**Methods:**

This was a control versus intervention study to evaluate the effect of fat grafting in the prevention on peritoneal adhesion formation. An experimental mouse model for moderate and extensive peritoneal adhesions was used (*n* = 4–6 mice/group). Adhesions were induced mechanically, and a free epididymal fat graft from wild type or CAG-DsRed mice was injected preperitoneally immediately after adhesion induction. PET/CT imaging and scaling of the adhesions were performed, and samples were taken for further analysis at 7 and 30 days postoperation. Macrophage phenotyping was further performed from peritoneal lavage samples, and the expression of inflammatory cytokines and mesothelial layer recovery were analysed from peritoneal tissue samples.

**Results:**

Fat grafting significantly inhibited the formation of adhesions. PET/CT results did not show prolonged inflammation in any of the groups. While the expression of anti-inflammatory and anti-fibrotic IL-10 was significantly increased in the peritoneum of the fat graft–treated group at 7 days, tissue-resident and repairing M2 macrophages could no longer be detected in the fat graft at this time point. The percentage of the continuous, healed peritoneum as shown by Keratin 8 staining was greater in the fat graft–treated group after 7 days.

**Conclusions:**

Fat grafting can inhibit the formation of peritoneal adhesions in mice. Our results suggest that fat grafting promotes the peritoneal healing process in a paracrine manner thereby enabling rapid regeneration of the peritoneal mesothelial cell layer.

**Electronic supplementary material:**

The online version of this article (10.1007/s11605-019-04425-4) contains supplementary material, which is available to authorized users.

## Introduction

Up to 93% of patients develop adhesions after intra-abdominal surgery.^1^ Adhesion formation can contribute to postoperative complications in abdominal and gynecological surgery such as chronic abdominal or pelvic pain, infertility, and intestinal obstructions. The clinical consequences of peritoneal adhesions have a significant economic impact as the treatment of peritoneal adhesions amounts to US$1.3 billion per year in the USA alone.^2^ Peritoneal adhesion formation results from abnormal healing after peritoneal trauma, and the key features of this process include harmful inflammation and unbalanced fibrinolysis.^3^ Mesothelial injury is considered to be one of the causes of peritoneal adhesion formation as the integrity of the mesothelial layer promotes fibrinolytic activity.^4, 5^

Free fat grafts and processed components of fat are used for reconstruction of soft tissue defects especially in the facial and thoracic region. Novel experimental therapies include softening and contouring of scars and other fibrotic conditions, e.g. in the perineal and anal region^6, 7^ and Dupuytren’s contracture.^8, 9^ Fat grafting has been shown to result in histological improvement of scars in terms of general structure, collagen remodeling and vascularisation.^10, 11^ However, little is known about the role of fat graft macrophages during this process although they are abundant in fat and contribute to tissue remodeling and wound healing.^12^

The prevention and treatment strategies for peritoneal adhesions have focused mainly on optimizing the surgical techniques and development of mechanical barriers to prevent contact of the damaged serosa and the adjacent organs. Although various treatment strategies have been investigated, the optimal solution for adhesion prevention still remains uncovered.

The wound healing process of the peritoneum resembles that of the dermal wound in many ways.^2^ As fat grafting prevents excessive dermal scarring, we hypothesized that it could also have an effect on abnormal peritoneal wound healing. The purpose of this study was to investigate the therapeutic potential of fat grafting in the prevention of peritoneal adhesions using a mouse model for moderate and extensive adhesions. We also hypothesized that the adhesion preventive effect of the fat graft could be mediated by the grafted macrophages. Therefore, we studied the effect of fat grafting on the inflammatory and regenerative status of the healing peritoneum in terms of inflammatory cytokine expression, macrophage infiltration, mesothelial cell regeneration and metabolic activity.

## Materials and Methods

### Animals

C57BL/6N male mice (age 8–12 weeks) were purchased from the Central Animal Laboratory of University of Turku. CAG-DsRed (in C57BL/6N background, stock 005441) mice that express DsRed in all cells were purchased from The Jackson Laboratory. All animal experiments were approved by the National Animal Experiment Board in Finland. They were carried out in adherence with the rules and regulations of the Finnish Act on Animal Experimentation (497/2013) under license number ESAVI/10829/04.10.07/2015.

### Mice Model for Adhesion Prevention—Moderate and Extensive Adhesions

Mice were anesthetized with 2.5% isoflurane/O_2_. For analgesia, buprenorphine hydrochloride (Temgesic, RB Pharmaceuticals Limited) was administered before the operation and on the first postoperative day at 0,075 mg/kg.

**Fig. 1:**
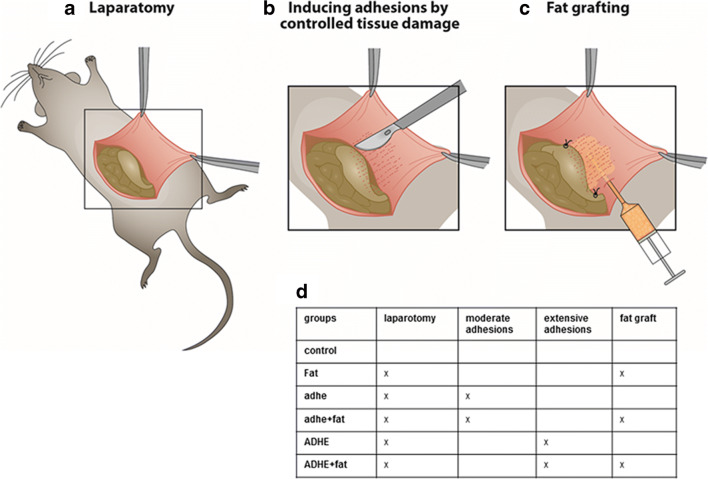
Overview of the surgical protocol. A midline incision was made through the abdominal wall and peritoneum (a). Standard sites of 0.5 × 1 cm of the cecum and peritoneal surfaces were scraped with a scalpel 30 times until hyperemia of the cecum wall vessels was noted. One 7-0 Prolene suture was used to suture the peritoneum and cecum together at the inferior margin of the injured site (group adhe). To induce more extensive adhesions, an area of 2 × 1 cm of the cecum and peritoneal surfaces was scraped and two 7-0 Prolene sutures were used to suture the peritoneum and cecum together at the inferior and superior margin of the injured site (group ADHE) (b). A small titanium clip was placed on the peritoneal surface 0.5 cm above the injured site to enable visualization during PET-CT imaging. Free epididymal fat grafting (0.2 ml) was performed beneath the peritoneal wall at the damaged area immediately after adhesion induction during the same operation (c) (groups adhe + fat, ADHE + fat, fat). Different experimental groups are shown in d; all study groups contain six mice (*n* = 6) (d)

A midline incision was made through the abdominal wall and peritoneum. Standard sites of 10 × 5 mm of the cecum and peritoneal surfaces were scraped with a scalpel 30 times until hyperemia of the cecum wall vessels was noted. One 7-0 Prolene (Ethicon, NJ) suture was used to suture the peritoneum and cecum together at the inferior margin of the injured site (group adhe). For extensive adhesions, an area of 20 × 10 mm of the cecum and peritoneal surfaces was scraped and two 7-0 Prolene sutures were used at the inferior and superior margins of the injured site (group ADHE) (Fig. 1a–c). Epididymal fat grafts (0.2 ml) were injected beneath the peritoneal wall at the damaged area (groups adhe + fat, ADHE + fat, fat) using a 19 G fat grafting cannula (Tulip, CA). Preperitoneal injection was used to target the submesothelial layer of the peritoneum, which contributes to peritoneal healing. In the fat graft control group (group fat), a sham laparotomy was performed and fat grafting was performed as above without adhesion induction. The midline incision was closed in two layers using 7-0 Prolene and 5-0 Dafilon (B. Braun Melsungen, Germany) uninterrupted sutures. All study groups contained six mice (Fig. 1d). For macrophage phenotyping experiments, the 7-day time point and extensive adhesion model (ADHE) was used; these study groups contained four to six mice.

### Harvesting and Preparation of the Epididymal Fat Graft

Syngeneic C57B1/6N or CAG-DsRed donor mice were sacrificed, and white adipose tissue from intra-abdominal epididymal fat pads was collected. Two hundred μl of tissue was obtained from one epididymal fat pad site. CAG-DsRed mice were only used in 7-day time point macrophage studies. Fat pads were mechanically disrupted with scissors, and 50 μl of sterile saline was added to the suspension. Thereafter, homogenization was continued by repeatedly passing the fat suspension through a syringe and a 19 G needle. Fat decantation was performed for 15 min, and excess fluid was discarded.

### In Vivo ^18^F-FDG PET/CT Imaging and Data Analysis

Metabolic activity of the adhesions and fat grafts was examined after 30 days by PET/CT (Siemens Medical Solutions USA, Knoxville, TN) using ^18^F-FDG, a tracer known to accumulate in metabolically active tissues. The two-step whole-body PET/CT scan was performed during isoflurane gas anesthesia. ^18^F-FDG (approx. 5 MBq) was injected intravenously 120 min before the PET scan. Mice were placed in the central field of view of the scanner, and the scan was launched with a low-resolution 10-min CT scan used for attenuation correction, followed by a 20-min static emission PET scan. ^18^F-FDG images were reconstructed as described by Tervala et al.^13^; see Supplementary Material for details.

### Absolute Radioactivity Analysis of Tissue Samples

Immediately after the PET/CT imaging at 30-day time point, mice were humanely sacrificed for ex vivo tissue sample analysis. Blood samples were collected by means of cardiac puncture. Samples of the peritoneum and fat grafts were collected and measured for ^18^F-radioactivity in a single-well counter of an isotope calibrator and expressed as the percentage of the injected dose per gram (% ID/g) tissue of ^18^F-FDG-derived radioactive compounds.

### Adhesion Scaling

#### Moderate Adhesions (adhe)

Adhesions were scaled by a gastrointestinal surgeon blinded to the experiment. The adhesion scale was modified from Nair et al.^14^ Adhesions were scaled macroscopically right after sacrifice and from photographs according to the length of the adhesion area (0 = no adhesions, 1 = 1–2 mm, 2 = 3–5 mm, 3 = over 5 mm) as well as adhesion tenacity (0 = no adhesion, 1 = release with gentle pulling with forceps or by just touching, 2 = release with blunt dissection, 3 = needs to be cut with scissors). The adhesion length and tenacity were added to generate a total score.

#### Extensive Adhesions (ADHE)

Adhesions were scaled macroscopically according to the length of the adhesion area (0 = no adhesions, 1 = less than 10 mm, 2 = 10–15 mm, 3 = over 15 mm) as well as adhesion tenacity (scale same as above).

### Histological Analysis

Block resection tissue samples of the adhesion area were fixed in formalin for embedding in paraffin. Sections of 4-μm thickness were stained with haematoxylin and eosin and Wright van Gieson to visualize the connective tissue of the adhesion site and peritoneum. To evaluate the effect of fat grafting on the thickness of the connective tissue layer of the peritoneum, the thinnest and thickest parts of the peritoneal wall of each sample were measured independently by two researchers and the average of these was calculated. Inflammation was graded on a semiquantitative scale of 0 to 5 by evaluation of the relative presence of inflammation (as evidenced by infiltration of lymphocytes and macrophages) as follows: 0 = absence, 1 = minimal presence, 2 = minimal to moderate presence, 3 = moderate presence, 4 = moderate to extensive presence, and 5 = extensive presence. The same scale has been used by Tervala et al.^13^

### Immunofluorescence Staining

Four-μm-thick paraffin sections were deparaffinized and rehydrated. Staining was performed with primary antibodies against Collagen 1, Keratin 8, and a-smooth muscle actin (Acta2) diluted in 10% FBS-PBS, and fluorochrome-conjugated secondary antibodies (AlexaFluor488 anti-rabbit, AlexaFluor 594 anti-rat and AlexaFluor647 anti-mouse; Invitrogen), diluted in 10% FBS-PBS as previously described.^15^ Details are provided in the Supplementary Material. After incubation, the slides were washed with PBS and labelled with DAPI diluted 1:3000 in washing buffer for 5 min. Slides were rinsed once with PBS and mounted with Mowiol. Samples were viewed with 3i CSU-W1 spinning disk confocal microscope or Pannoramic Midi FL slide scanner (3DHISTECH), and the images were analyzed using Fiji Image software. The integrity of the peritoneal mesothelial layer was calculated as percentage of Keratin 8–positive mesothelium of the whole peritoneal surface of the samples.

Peritoneal samples were snap frozen in OCT (Tissue-Tek Sakura) and later stained with F4/80 for detection of macrophages. See Supplementary Material for specific protocol.

### RT-qPCR

RNA isolation from peritoneal wall and fat graft samples was performed applying TRI Reagent (MRC Inc.) according to the manufacturer’s protocol. After isolation, RNA was converted to cDNA using cDNA mix (Quanta Biosciences) according to the manufacturer’s protocol. Quantitative real-time PCR was performed with Rotor-Gene Q real-time instrument (Qiagen, Hilden, Germany) with primers for GAPDH, IL-10, TGF-b1, IL-1b, TNF-a, IL-2, IL-4, IL-12b, IL-13, and IL-17a. See Supplementary Material and Table S1 for protocol and primers. RT-qPCR results were obtained by the ΔΔCt method^16^ using GAPDH as a housekeeping gene; the median values are used. Statistical significances were determined from ΔCt values.

### Macrophage Phenotyping

For macrophage phenotyping experiments, the extensive adhesion model was used. Fat grafting from CAG-DsRed mice was performed as described above. Mice were sacrificed on day 7, and fat grafts and peritoneal cells were harvested by flushing the peritoneal cavity with RPMI 1640 supplemented with 2% FCS and 5 IU heparin/ml. Fixable live/dead cell staining was used according to the manufacturer’s instructions (Fixable Viability Dye eFluor 780; eBioscience). Before the antibody staining, the cell suspensions were incubated with purified anti-CD16/32 (clone 2.4G2; Bio X Cell BE0206) for 10 min on ice to block non-specific binding to Fc receptors. See supplements for list of antibodies. Stainings were performed in + 4 °C for 20 min. All FACS analyses were run using LSRFortessa flow cytometer (BD Biosciences) and analyzed using FlowJo (Tree Star Inc.) software.

### Statistical Analysis

Statistical analysis was performed using GraphPad Prism 7 software. One-way ANOVA and Tukey’s multiple comparison test, Mann-Whitney *U* test, or Kruskal-Wallis analysis of variance test followed by Dunn’s multiple comparison test were used to calculate statistical differences between groups depending on data type and normality. *p* < 0.05 was considered statistically significant.

## Results

Both moderate (adhe) and extensive adhesion (ADHE) groups were analysed for the adhesion score and PET/CT analysis 7–30 days postoperation, whereas only ADHE groups were analysed by histology, immunofluorescence, RT-PCR and flow cytometry 7–30 days postoperation.

### No Prolonged Inflammation Seen with ^18^F-FDG PET/CT

**Fig. 2:**
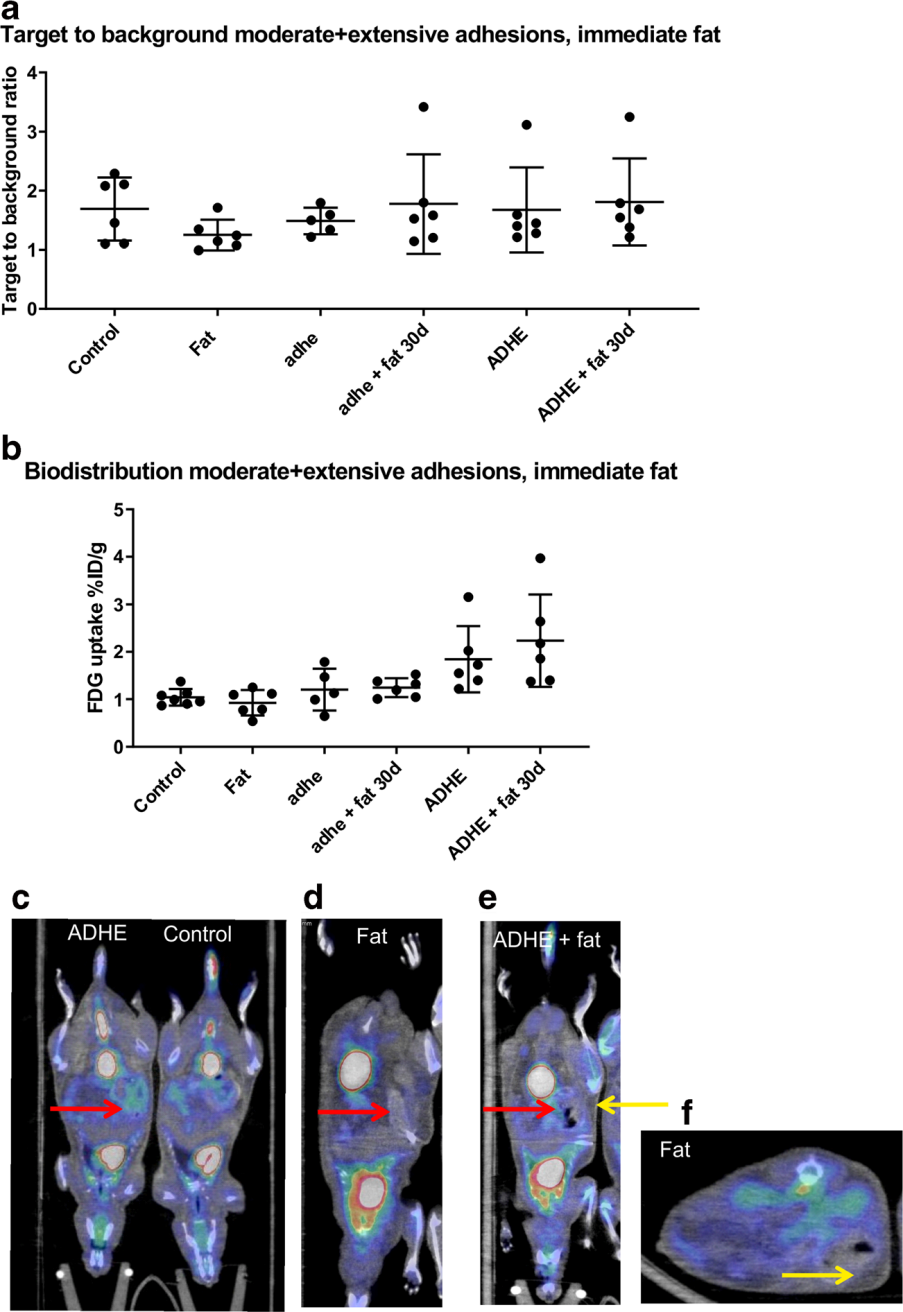
In vivo ^18^F-FDG PET/CT results presented as target-to-background (tissue-to-liver) ratio (**a**) and ex vivo biodistribution results presented as the percentage of injected dose per gram tissue (% ID/g) (**b**). There was a trend towards higher ^18^F-FDG uptake in the extensive adhesion groups when comparing absolute radioactivity of the samples, but no effect of the fat graft could be detected. Representative PET/CT images of **c** ADHE and control, **d** fat, **e** ADHE + fat, and **f** transverse plane of fat showing slightly increased FDG uptake in the adhesion area in ADHE and ADHE + fat groups. Red arrows point to area of adhesions, and yellow arrows point to fat graft (*n* = 6)

To examine if fat grafting influences the metabolism of the adhesion area, we analyzed the mice with PET/CT imaging and absolute radioactivity measurements after 30 days (expressed as tissue-to-liver ratio). No significant differences were observed between the experimental groups when comparing standardized uptake values (SUVs), and the metabolic status was also of similar magnitude in all of the groups (Fig. 2a). Though, a trend towards a higher ^18^F-FDG uptake in the ADHE groups was observed when comparing the absolute radioactivity. However, no effect of the fat graft could be detected on the metabolic rate (Fig. 2b). Representative PET/CT images show a slightly increased FDG uptake in the adhesion area in ADHE and ADHE + fat groups (Fig. 2c–f).

### Adhesion score is lower in the fat graft treated group in the moderate adhesion model

**Fig. 3:**
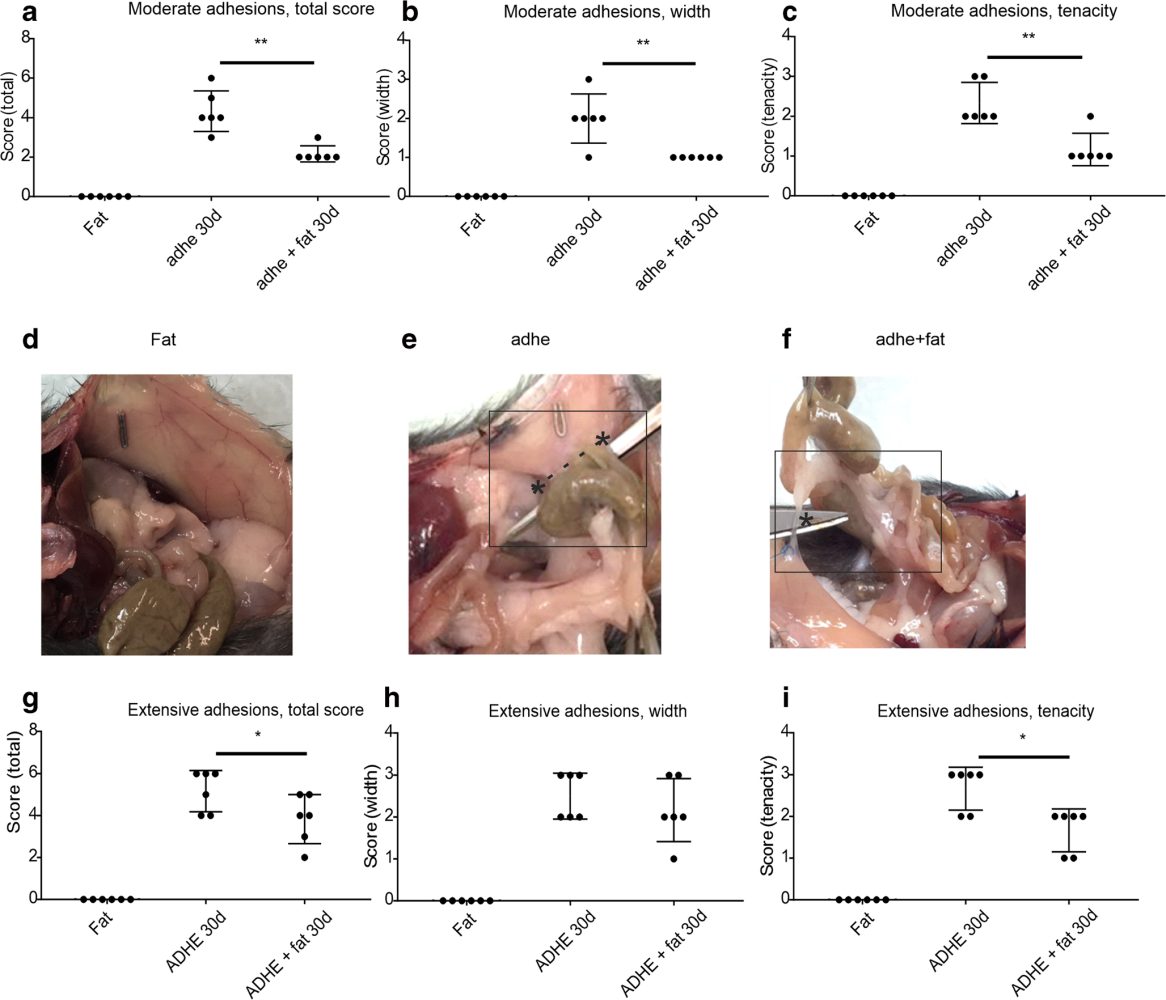
Fat graft inhibits adhesion formation in a moderate adhesion model (**a**–**f**). Intra-abdominal adhesion scores (total (**a**), width (**b**), tenacity (**c**)) 30 days after moderate adhesion induction by a cecum scraping model and fat grafting. Data presented as mean ± SD, *p < 0.05, **p < 0.01. Fat = fat graft only, adhe = moderate adhesion induction, adhe + fat **=** adhesion induction + fat graft (*n* = 6). Representative images of adhesions on day 30: fat (**d**), adhe (**e**), adhe + fat (**f**); * pointing the location of adhesion. Fat graft inhibits adhesion formation in an extensive adhesion model (**g**–**i**). Intra-abdominal adhesion scores (total (**g**), width (**h**), tenacity (**i**)) 30 days after extensive adhesion induction by a cecum scraping model and fat grafting. Data presented as mean ± SD, *p < 0.05. Fat = fat graft only, ADHE = extensive adhesion induction, ADHE + fat **=** adhesion induction + fat graft (*n* = 6)

When comparing the moderate adhesion groups, the adhesion score was significantly lower in fat graft–treated mice at 30-day time point (4.3 ± 1.0 in adhe group and 2.2 ± 0.4 in adhe + fat group, *p* < 0.01) (Fig. 3a). Both adhesion width and tenacity were lower after fat grafting (*p* < 0.01) compared with the adhesion control group (Fig. 3b, c). In the fat graft control group (fat) (without adhesion induction), no adhesions were present (Fig. 3d). In moderate adhesion control group, most adhesions were tight and needed lysis to be released (Fig. 3e). In fat graft–treated group, the adhesions that were present were film-like and easily released (Fig. 3f). As peritoneal trauma is known to be an inductor of adhesions, it is very unlikely that the injection itself (sham graft) would be the cause of reduced adhesion formation, as the injection is a small trauma.

### Adhesion score is lower in the fat graft treated group in the extensive adhesion model

In the extensive adhesion group, the adhesion score was also significantly lower at 30-day time point in mice that had been treated with the fat graft (5.2 ± 1.0 in ADHE group and 3.8 ± 1.2 in ADHE + fat group, *p* < 0.05) (Fig. 3g). Adhesion tenacity but not width was significantly lower after fat grafting (*p* < 0.05) (Fig. 3h, i). Interestingly, adhesion scores were significantly lower in the fat graft–treated group already 7 days after induction of extensive adhesions (3.75 ± 0.5 in ADHE group and 2.6 ± 0.52 in ADHE + fat group, *p* < 0.05, data not shown).

### Peritoneal Trauma Induces Peritoneal Thickening and Fat Grafting Promotes Mesothelial Cell Regeneration

**Fig. 4:**
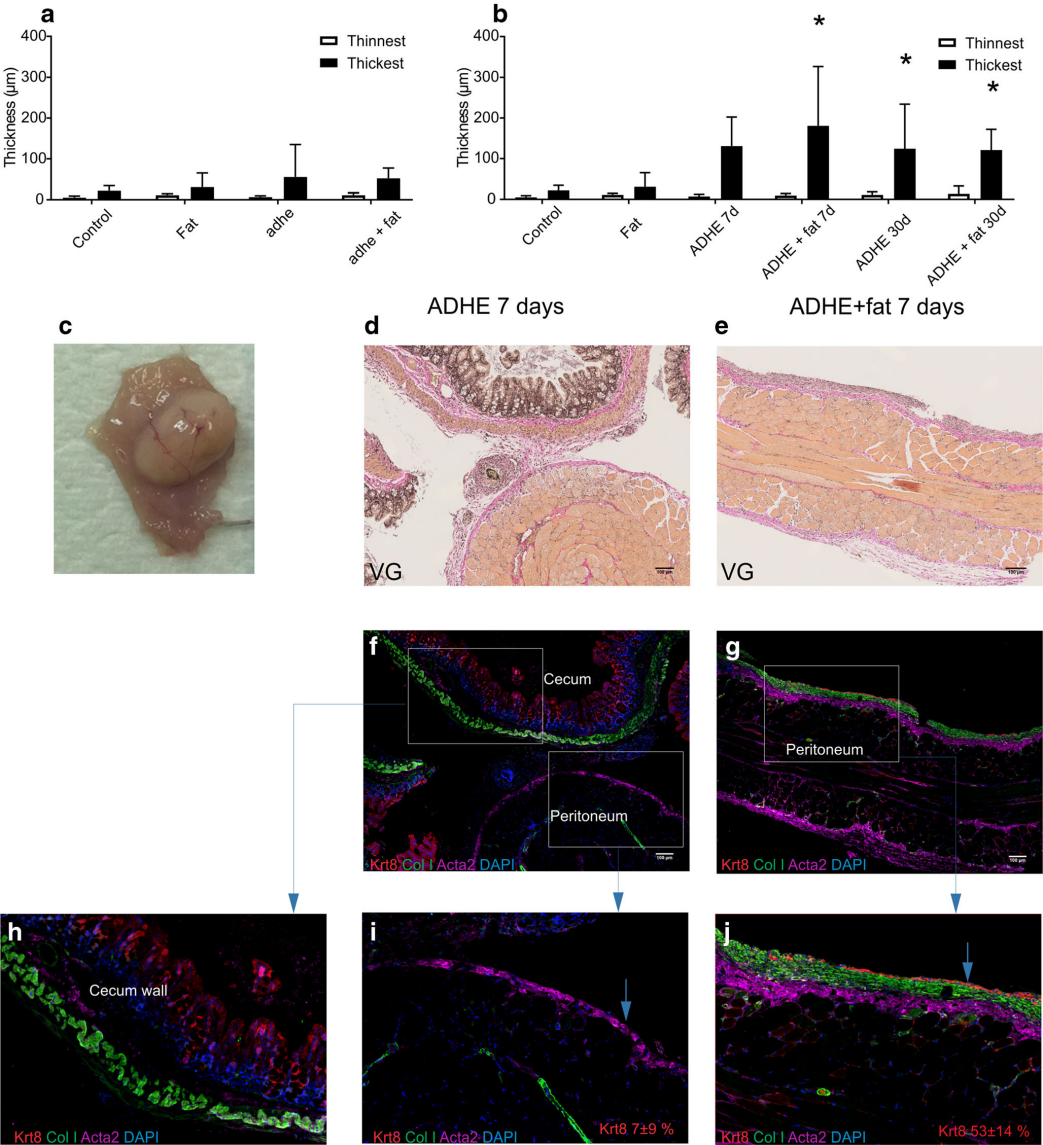
Peritoneal wall thickness at 30 days in the moderate adhesion model (a). Peritoneal wall thickness at 7 and 30 days in the extensive adhesion model (**b**). The thinnest and thickest parts of the peritoneal wall were measured from each sample independently by two researches, and the average of these measurements was calculated. *p < 0.05 compared with the control group. Representative image of fat graft at 30 days (**c**). Wright von Gieson staining (**d**, **e**) and immunofluorescence staining of Collagen I, Acta2, Keratin 8 and DAPI of the injured peritoneal surfaces at 7 days (**f**, **g**). Higher magnifications showing Keratin 8 staining on the epithelial surface of the cecum lumen (**h**) but not on the peritoneal surface of ADHE samples (**i**). A more continuous Keratin 8 layer was detected on the ADHE + fat samples (**j**) The mesothelial layer integrity (as visualized by the Keratin 8 staining) was calculated as percentage of the positive Keratin 8 staining of the peritoneal surface from the whole sample. Keratin 8 staining was 7 ± 9% in the ADHE samples and 53 ± 14 % in the ADHE + fat samples (mean ± SD). Arrows point to the peritoneal surface layer (**i**, **j**)

The thickness of the peritoneal connective tissue layer was increased after extensive adhesion induction at 7 and 30 days (*p* < 0.05 vs. control group) (Fig. 4a, b, d, e). Fat grafting itself did not induce peritoneal thickening, and no significant differences in peritoneal thickness could be observed between ADHE vs. ADHE + fat groups (Fig. 4a, b, d, e). At 7 days, there were no differences in peritoneal inflammation and macrophage scores (inflammation ADHE 2.3 ± 2 vs. ADHE + fat 2.3 ± 0.9 and macrophage score ADHE 2.3 ± 2 vs. ADHE + fat 2.3 ± 1.4 on a scale of 1–5; data not shown). The adhesion tissue was so thin and film-like in the fat graft–treated groups that it was not possible to get proper samples of the adhesive tissue itself. At 30 days, fat grafts could be seen as a ball-like mass rather than the fan shape they were injected in (Fig. 4c).

Extracellular matrix and smooth muscle layer in the healing peritoneum were labelled with Collagen I and Acta2 antibodies. Keratin 8 labelling was used to observe the integrity of the mesothelial cell layer of the injured peritoneum. In the ADHE + fat samples, the percentage of the continuous healed mesothelium was greater than in the ADHE samples (ADHE + fat 53 ± 14% vs ADHE 7 ± 9%) (Fig. 4f–j).

### Fat Grafting Combined to Peritoneal Trauma Modifies Inflammatory Cytokine Gene Expression

To compare the temporal expression of inflammatory cytokines, RT-PCR of peritoneal wall samples was performed at 7 days and 30 days postoperation. Overall, fat grafting without adhesion induction (fat) induced a trend towards downregulation of many inflammatory genes compared with the non-operated control.

**Fig. 5:**
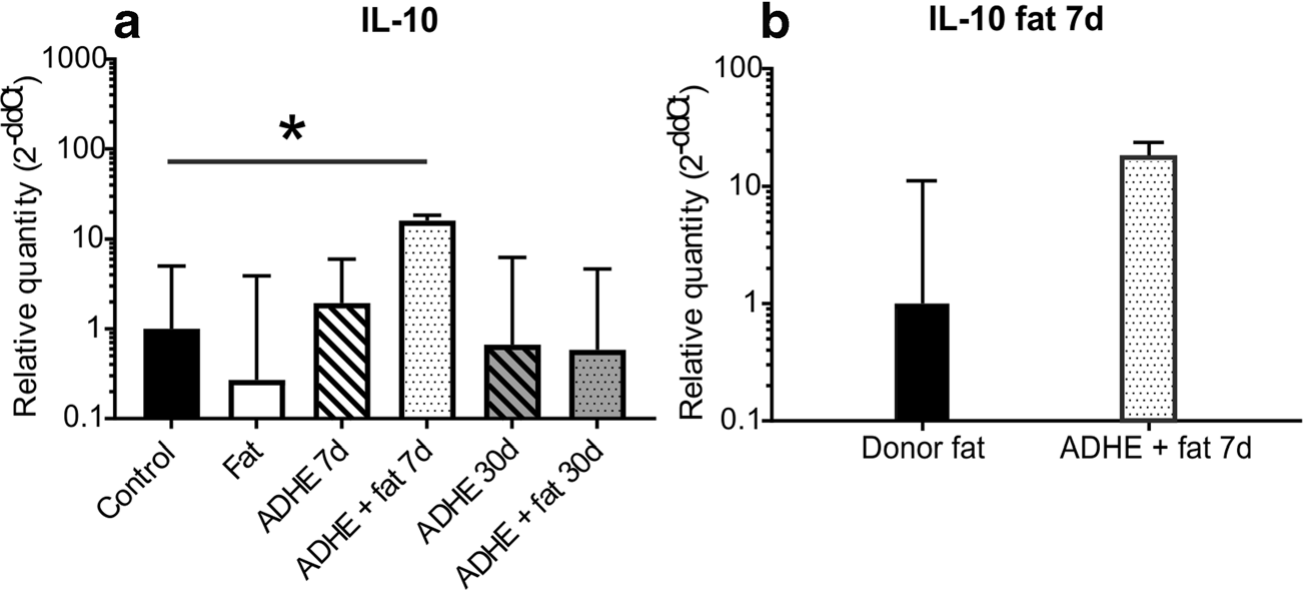
Expression of IL-10 (**a**) mRNA in peritoneal wall samples of control, fat ADHE and ADHE + fat groups at 7 and 30-day time points. Expression of IL-10 (**b**) in donor fat and fat graft after 7 days of grafting in ADHE + fat group, *p* < 0.05 compared with the control group (*n* = 4–6)

Interestingly, the expression of the anti-inflammatory cytokine IL-10 was significantly increased at 7-day time point in the ADHE + fat group compared with non-operated control (Fig. 5a). IL-10 expression in the grafted fat was also elevated compared with the donor fat at 7 days, but this difference was not significant (Fig. 5b). The other investigated cytokines, TNF-a, IL-2, IL-12, IL-17, IL-4, and TGF-b1, did not exhibit statistically significant changes in expression between the groups, although elevated expression levels of TNF-a, IL-2, IL-12, and IL-17 were observed in ADHE + fat group compared with the control group and ADHE group (Fig. S1).

### M2 Macrophages Disappear from the Fat Graft but Do Not Migrate to the Peritoneal Wall or Cavity

**Fig. 6:**
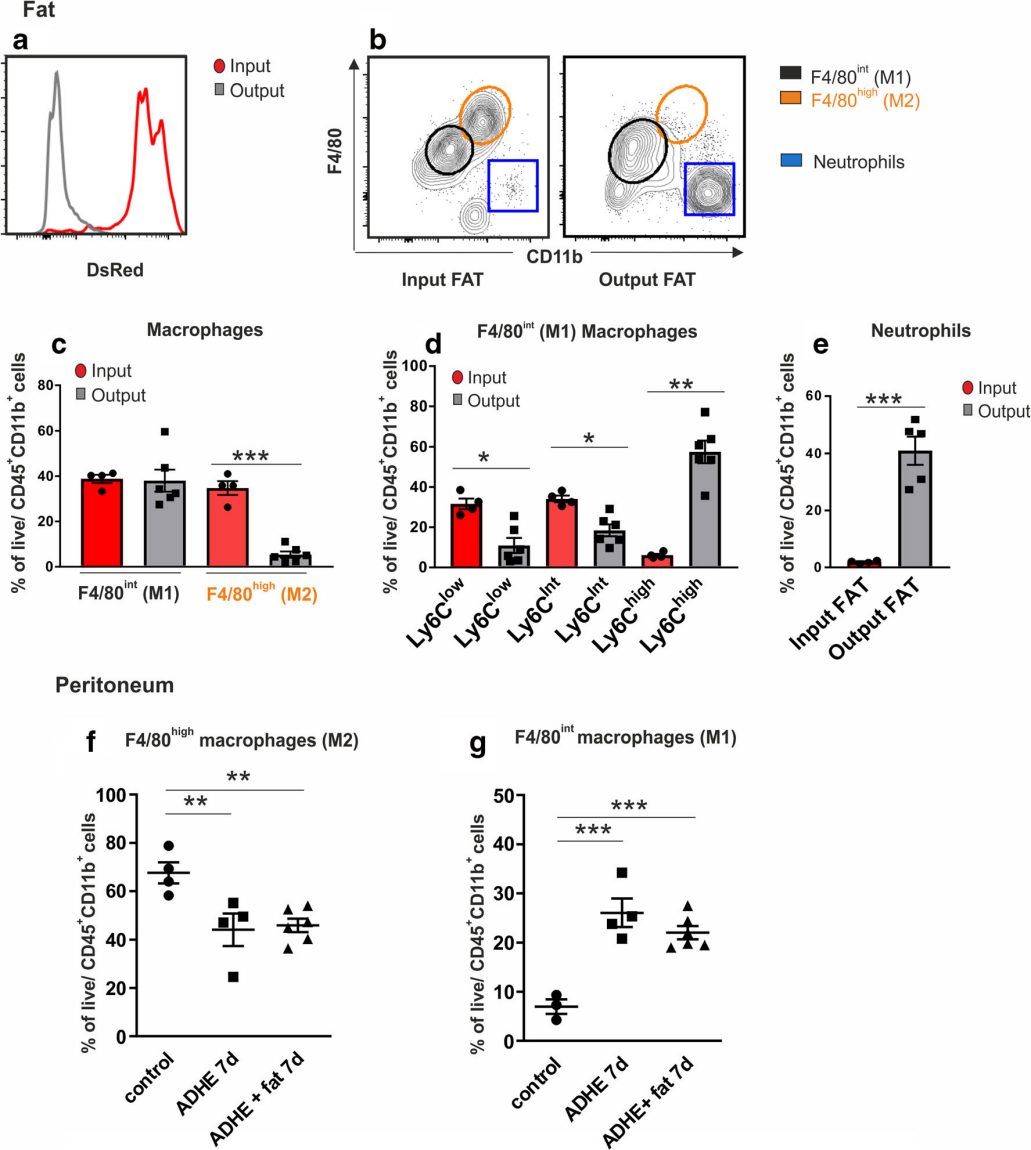
Flow cytometric analysis of macrophage and neutrophil populations in the input and output fat (**a**–**e**) and peritoneal cavity (**f**–**g**). Fat from DsRed fluorescent mice was used for grafting to allow tracking of donor (DsRed+) and recipient (DsRed−) mouse cells. Output fat population includes both DsRed-positive and DsRed-negative cell types. The flow cytometric data are shown as frequency of live-gated CD45+CD11b+ cells (all leukocytes). F4/80 expression intensity is used to divide macrophages into tissue-resident (M2) and bone marrow–derived (M1) inflammatory macrophages. Each dot represents one mouse (pooled from two independent experiments), and the bars represent the mean ± SEM of each group (*p < 0.05 **p < 0.01 ***p < 0.005 vs control). Seven-day time point and extensive adhesion model are used; control = non-operated control (C57BL male mice); study groups contain four to six mice

To investigate the graft-derived macrophage population in the peritoneum, fat from mice with ubiquitous DsRed expression was used for grafting (Fig. 6a). The fat grafts and peritoneal lavage samples were analyzed at 7 days.

In the grafted fat samples (input fat), a large population of CD11b^high^ F4/80^high^ tissue-resident macrophages (which typically have an anti-inflammatory M2 phenotype) were present accompanied by a population of CD1lb^int^ F4/80^int^ bone marrow–derived macrophages (which typically represent monocyte-derived pro-inflammatory M1-like cells) (Fig. 6b, c). During the 7 days of fat grafting, the tissue-resident M2 macrophages had almost completely disappeared (input vs. output FAT, *p* < 0.001) (Fig. 6c). The high frequency of Ly6C^high^ cells (Ly6C is a canonical marker of inflammatory blood monocytes, which is downregulated in macrophages) among the myeloid cells in the fat after 7 days indicates that there had been an influx of blood-derived monocytes to the fat graft (Fig. 6d). This is in line with the observation that the output fat was infiltrated with a new myeloid population which was not DsRed expressing indicating that the myeloid cells were derived from the recipient mice (Fig. 6a, c). Also a large population of neutrophils was recruited to the fat grafts during the 7 days (input vs. output FAT, *p* < 0.001) (Fig. 6e).

In the non-operated control mice, the macrophage population in the peritoneum consisted mainly of M2 macrophages. After peritoneal trauma, the M2 macrophage population was slightly diminished (Fig. 6f) (control vs. ADHE, *p* < 0.01, control vs. ADHE + fat *p* < 0.01), while a significant M1 population appeared (Fig. 6g) (control vs. ADHE *p* < 0.005, control vs. ADHE + fat *p* < 0.005). This effect was slightly smaller in the fat graft–treated group, although no significant differences between the ADHE and ADHE + fat groups could be observed (Fig. 6g). Furthermore, all macrophages in the peritoneal cavity samples were DsRed negative indicating their origin from the recipient mice itself and not from the DsRed fat graft.

We also investigated the macrophage population of the peritoneum using immunofluorescence staining to see whether the disappeared M2 macrophages from the fat graft would have migrated into the peritoneum. However, no DsRed-positive macrophages were visible in the peritoneum of the mice at 7 days after fat grafting (data not shown).

## Discussion

Our study provides the first evidence that preperitoneal fat grafting reduces peritoneal adhesion formation in a mouse model. Our results demonstrate that fat grafting prevents peritoneal adhesion formation, both in the moderate and extensive adhesion models. The effect was most prominent in adhesion tenacity. We also observed that fat grafting increased the expression of the anti-inflammatory cytokine IL-10 in the ADHE + fat group compared with the non-operated control group. Interestingly, fat grafting seemed to promote faster mesothelial healing of the peritoneum.

Although various novel strategies have been investigated for treatment of adhesions,^5, 17^ the optimal solution remains uncovered. When comparing treatment strategies, fat grafting has several advantages: it appears anti-inflammatory and anti-fibrotic and provides tissue-resident repairing or resolving macrophages to the damaged area.^18–21^ Cil and Aydogdu hypothesized that autologous fat grafting boosts remesotheliazation and decrease adhesions.^22^ Formation of adhesions is a complex cascade with many factors involved, including haemostasis, inflammation, peritoneal wound healing, mesothelial regeneration and extracellular matrix production. Instead of just blocking one pathway or using mechanical contact prevention of the peritoneal surfaces, fat grafting can target many of these factors simultaneously. Originally, we also tested intraperitoneal placing of the fat graft but found that after 7 days, there was nothing left of the graft as it was probably cleared by the intraperitoneal macrophage population because it was freely moving. Preperitoneal injection is a novel way to target peritoneal healing as most treatment options are placed intraperitoneally.^2^ The stromal cells in the submesothelial layer of the peritoneum contribute to peritoneal wound healing,^23^ and the preperitoneal injection targets this layer. In future studies, we plan to investigate which component of the fat tissue is responsible for the effect and whether the effect is seen only locally or also further from the injected area. If the effect is caused by a certain cell type or combination of cells (for example, the stromal vascular fraction), injection could be made in the preperitoneal space and also intraperitoneal space. This way, it could target the inter-loop adhesions as well without the risk for fat cell necrosis. Also, the preperitoneal space in humans is looser than in mice, and the injection might spread to a greater area. However, these matters warrant future research. Our results clearly show a reduction in the adhesion score after fat grafting in mice, which is a novel and clinically relevant finding.

Fat or its components, including stromal vascular fraction (SVF) or adipose derived stem cells (ADSC), have been shown to affect many inflammatory parameters. They have been shown to reduce pro-inflammatory cytokine (IL-1b, IL-17, TNF-a) levels and increase anti-inflammatory protein (IL-10) levels in mice at 4–10-week time points.^18, 19, 21^ Although TGF-b- is a major regulator of wound healing and fibrosis,^2^ we found no significant differences in TGF-b1 expression between the experimental groups. It has also been shown that IL-10 can reduce scar formation and fibrosis by inhibiting excessive deposition of extracellular matrix and by regulating the arrangement of collagen fibres in regenerated tissue.^24, 25^ IL-10 is also known to protect from TGF-b-induced fibrosis.^24, 25^ IL-10 is produced by various cell types, including Th2 cells, regulatory T cells and macrophages.^12^ Furthermore, Holschneider et al. found that intraperitoneal IL-10 treatment reduced the formation of peritoneal adhesions in a rat model.^26^ Our results demonstrate a significantly increased RNA expression of anti-inflammatory IL-10 in the fat graft–treated peritoneal trauma group than in the control mice, supporting a potential role for IL-10 in reduced formation of adhesions. Further, greater IL-10 expression could guide the wound healing process faster to the proliferative and remodelling phases.^12^ The PET/CT results showed that metabolic activity was of similar magnitude in all groups. This suggests that our experimental model does not involve sustained inflammation at the 30-day time point in any of the groups.

We looked closer at the structure of the peritoneal surface and found that in the fat graft–treated mice, the integrity of the mesothelial cell layer seemed to be more complete after 7 days compared with the untreated group. This suggests that fat grafting may promote faster mesothelial regeneration after injury.

Macrophages have a major role in orchestrating the wound healing process. Typically, the tissue-resident macrophages are thought to contribute heavily to the M2-like macrophage pool, which is involved in anti-inflammatory and tissue repair responses. The pro-inflammatory M1-like macrophages, in contrast, are usually largely derived from the adult monocytes and heavily infiltrate tissues under inflammatory conditions^12^. Our results show that the M2 macrophages disappear from the fat graft, which has not been previously reported. The direct cellular effect of the fat graft, however, remains to be investigated, as we could not detect fat graft-derived macrophages in the peritoneum or peritoneal cavity. Cells of the SVF, which include all the other cells of fat besides adipocytes, have been shown to promote activation of tissue repair macrophages in a different type of inflammatory model^20^. Thus, a paracrine effect of the fat graft, including but not limited to IL-10, is a potential mechanism for reduction of adhesion formation, but confirming this hypothesis requires further investigation.

Fat grafting as such can result in bulkiness of the area as it is also used for volume increase purposes. In future studies, the therapeutic potential of SVF, macrophages and adipose-derived stem cells as individual components could be compared with that of the fat graft. As a conclusion, our study shows that fat grafting can prevent peritoneal adhesion formation in mice, which opens up novel areas of research for peritoneal adhesion prevention.

## Electronic supplementary material

ESM 1 (TIF 20389 kb)

High resolution image (PNG 690 kb)
