# Supplementary figures and images for: Preperitoneal Fat Grafting Inhibits the Formation of Intra-abdominal Adhesions in Mice
Source: J Gastrointest Surg. 2019 Dec 10;24(12):2838–48. doi: 10.1007/s11605-019-04425-4 (PMC7674570; doi:10.1007/s11605-019-04425-4)

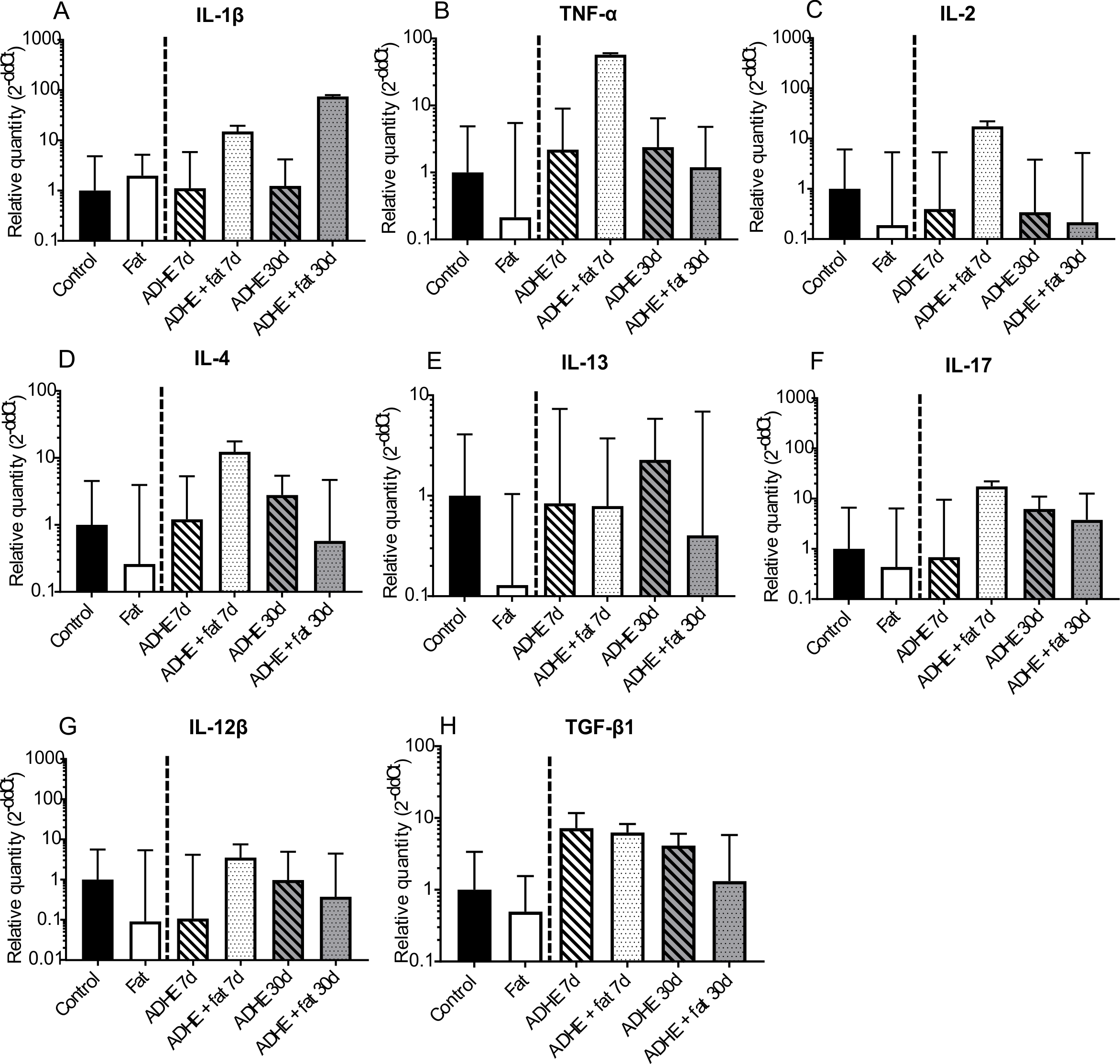

Supplement: Supplementary file 1 — (TIF 20389 kb) [file 11605_2019_4425_MOESM1_ESM.tif]

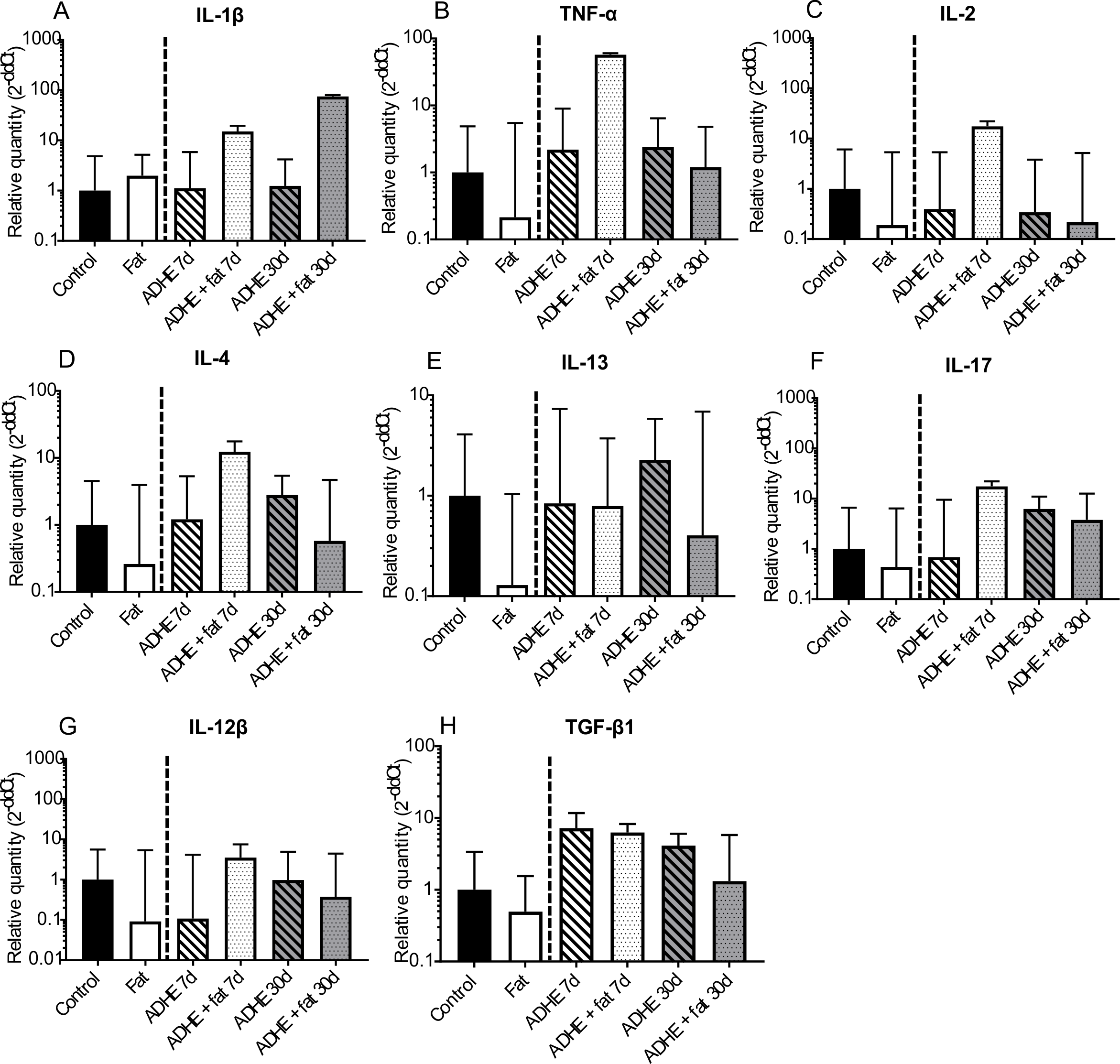

Supplement: Supplementary file 2 — High resolution image (PNG 690 kb) [file 11605_2019_4425_Fig7_ESM.png]
